# Circumcision Complications Associated with the Plastibell Device and Conventional Dissection Surgery: A Trial of 586 Infants of Ages up to 12 Months

**DOI:** 10.1155/2008/606123

**Published:** 2008-11-04

**Authors:** Seyed Abdollah Mousavi, Ebrahim Salehifar

**Affiliations:** ^1^Department of Pediatric Surgery, Mazandaran University of Medical Sciences, Sari 48157 33971, Iran; ^2^Department of Clinical Pharmacy, Faculty of Pharmacy, Mazandaran University of Medical Sciences, Sari 48157 33971, Iran

## Abstract

Conventional dissection surgery (CDS) or using the Plastibell device (PD) is the method most frequently employed for circumcision. The aim of this study was to evaluate two methods in terms of the incidence of complications in infants of ages up to 12 months. In a prospective study, 586 infants equal to or less than 12 months were studied from 2002 to 2008, and complications between the two groups were assessed. The overall rates of complications in CDS and PD groups were 1.95% and 7.08%, respectively. In each group, the rate of complications was not different among children who had a normal weight, compared to those of a lower or upper (10%) weight. There was a significant positive correlation between the age and weight of subjects within the time of ring separation (*P* < .001). The results of this study suggest the PD method for neonates and low-weight infants with thin prepuce and the CDS for other infants.

## 1. INTRODUCTION

Male circumcision has been performed for more than 5000 years [1] as a way to remove the redundant foreskin in order to expose the glands. About 25% of the total male population are circumcized, and circumcision remains one of the most common operations performed all over the world [2]. Over 60% of male newborns were circumcized in USA in 1992 [3]. In our country, all Muslim boys are ritually circumcized between the neonatal periods through the age of 4 to 5 years. The benefit of circumcision has been described in numerous studies, such as in the reduction risk of penile cancer [2], cancer of the cervix uteri [4, 5], urinary tract infections (UTIs) [6, 7], sexually transmitted diseases (STDs), and lower HIV prevalence [2, 6]. There are many procedures for circumcision; conventional dissection surgery (CDS) or using the Plastibell device (PD) is one of the methods most frequently employed for circumcision. The technique of choice remains controversial as we found only two published prospective randomized trials of circumcision in children, comparing the PD to a conventional dissection technique [8, 9]. These trials were performed 14 to 27 years ago, in which most children were older than infantile. On the other hand, there exist several reports of complications associated with the use of the PD in children circumcision [10, 11].

The aim of this study was to compare the various complications of two methods of circumcision in infantile age.

## 2. MATERIALS AND METHODS

This study was conducted on 586 children equal to or less than 12 months, who were brought by their parents for circumcision in an outpatient clinic, between September 2002 and January 2008. The study was approved by the Ethics Committee of Mazandaran University of Medical Sciences. All participants were full-term healthy males without any medical indication or urological anomaly, and had been operated on by one of our pediatric surgeons.

Informed consent was obtained from parents of eligible infants based on entry criteria. Infants were randomly divided by one of two techniques: the Plastibell method or conventional dissection (sleeve resection). We randomized infants in one of two groups unless the parents insisted on a particular circumcision method. Consequently, the number of subjects in Plastibell method was more than that of the conventional group. Infants were not fed for 1-2 hours prior to the procedure. After placing an infant on a circumcision restraint board, the skin was prepared with povidone iodine (10%) solution. A dorsal nerve block was administered using 0.2 mL/kg of 2% lidocaine, with a 27-gauge needle. Regardless of the technique, four minutes were allowed to elapse for all infants before beginning of circumcision procedures. In Plastibell technique, a plastic protective bell was placed over the glands and under the foreskin. A suture was placed around the entire foreskin, which would eventually fall off, after necrosis within several days. The parents of subjects were informed to return if the time of bell separation exceeded more than 10 days.

In the second group, a dissection suturing technique was used. After a circumferential incision along the line of the coronal sulcus, the foreskin was retracted to expose the glands. Then, a second circumferential incision was provided 1.5 cm proximal to the coronal sulcus. The foreskin was then carefully excised and the wound was closed with a 4/0 chromic. No dressing was applied in Plastibell method; however, a mild compress dress was used to prevent bleeding in the conventional dissection group.

Acetaminophen drop was used as an analgesic for children in both operations. In addition, parents were directed to do sits bath with soapy water twice per day, and also to apply a liberal amount of ophthalmic ointment gentamicin to the operative site for a period of ten days. All children were followed up until the wound was healed, along with observing them for any associated complications. The complications are, for example, infection, bleeding or hematoma, excess mucosa, bell disposition (entrapping the ring), and delayed falling.

Data were analyzed by SPSS 11.5 software, and *P*-value of <.05 was considered as a significant difference. The frequency of complications between two groups was assessed by chi-square test. Correlations between age and weight of cases with the separation time of the Plastibell method were investigated by Pearson correlation test.

## 3. RESULTS

The demographic characteristics of subjects are presented in Table 1. The mean age of both groups was less than 6 months. Considering the age and weight of the children, more than 90% had a normal weight. There was no significant difference between the two groups in terms of numbers of subjects who were in the upper or lower 10th percentile curves for weight.

Complications of circumcision by CDS and PD are presented in Table 2. The overall complication rates in CDS and PD groups were 1.95% and 7.08%, respectively.

In conventional dissection group, bleeding was the only complication. There were 6 infants who had continuous oozing. Two of them were hemophilic, and thus they were excluded from this study. The bleeding of four other infants stopped with compress dressing.

In Plastibell method, delayed separation of ring was the most common complication (2.6%) followed by bleeding, excess mucosa, infection, disposition, and hematoma. There were 10 infants whose bell did not separate after the 10th day; therefore, we removed the cup accordingly by cutting the tie. Infection was a clinical diagnosis and was not confirmed by culture. All subjects subsided without any adverse effects. Eight of subjects, who had bleeding, hematoma, or disposition of ring, were managed by reoperation and suturing.

There was a trend for significant difference regarding the rate of complications between PD and CDS (*P* = .051). In each group, the rate was not different among children who had a normal weight, compared to those who had lower or upper 10% weight.

**Figure 1:**
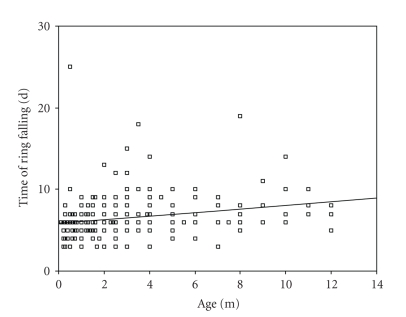
Correlation between age of subjects and the bell separation time (Pearson correlation = 0.24, *P* < .001).

**Figure 2:**
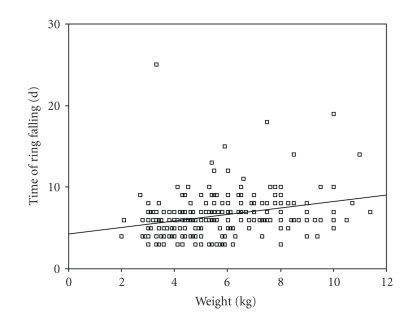
Correlation between the weight of subjects and the bell separation time (Pearson correlation = 0.29, *P* < .001).

Correlation between the age and weight of children in Plastibell group within the separation time of ring is demonstrated in Figures 1 and 2. There was a significant positive correlation between the age and weight of subjects within the time of ring separation (*P* < .001). This indicates that the ring separated faster in younger children. Eventually, the average procedure time for CDS and PD methods (in spite of the time needed for anesthesia) was 9.2 and 3.4 minutes (*P* < .01).

## 4. DISCUSSION

Routine neonatal circumcision can be a safe procedure [10]. The overall complication rate of the procedure ranges between 0.19% and 3.1% [12]; however, in a few studies, it was extremely high. Upon a retrospective study, Linus reported 20.2% complication in infants [12]. The less complication rate (17.6%) was reported in other randomized trials of childhood subjects [9].

Although many techniques for circumcision have been studied extensively [7], there are few reports determining which surgical technique may be associated with the least complications [8, 9].

A number of studies proposed that circumcision with PD is a simple method and complications including hemorrhage, local infection, sepsis, metal ulceration, and poor cosmetic results are rare [10, 11, 13]. On the other hand, tragic complications such as traumatic amputation of the glands and urethra-coetaneous fistula in CDS have been reported in other studies [14–18].

Mak et al. reported that the overall complication rates (intra- and postoperative) were similar between the conventional dissection and PD groups being 17.6% and 17.8%, respectively [9].

In a randomized trial study, Fraser et al. compared these two methods in childhood and concluded that the PD procedure is a satisfactory method for circumcising children [8].

Although comparison of these two circumcision methods has been reported in previously mentioned trials, as well as known ones, our study is unique in terms of the number of subjects who were less than or equal to 12 months of age, and the procedures for all subjects were performed by only one pediatric surgeon [8, 9].

PD is the most common technique used for neonatal circumcision around the world [1]. However, in our country the surgeons usually prefer conventional dissection methods. Fraser et al. had shown that the PD was a satisfactory method for circumcision of children up to the age of 8 years [8]. In our trial, we found that the overall complication rate of conventional surgical method was less than that of the Plastibell method (1.95% versus 7.08%). Although the *P*-value of complication comparison between PD and CDS groups was a little more than .05, the hazard ratio is so high (7.08/1.95 = 3.6).

The main complication associated with the PD in our study was the delayed separation of ring (2.6%). It should be noted that the ring separates faster in younger children due to thin prepuce and easier sloughing. Considering the lowest incidence of this complication in younger and thin infants, it would be considered a satisfied method for them.

There were eight cases whose bell was separated by a surgeon as an emergency setting. Six of them had bleeding or hematoma, while two cases had bell disposition.

Choosing the correct size of the Plastibell and close attention to ensure the ligature are sufficiently tied in order to prevent bleeding. In our study, bleeding was the second most common complication consisting of 18%. Lazarus et al. reported that bleeding was 44% from their observed complication [10].

The infection rate was 1% in Plastibell group, while no infant in the other group had infection. This is significantly lower than those reported by Mak et al. [9] (13.7% in Plastibell and 14.9% in dissection group), Fraser [8] (4% with both techniques), and Sorensen (5% with PD method) [19]. Since the criteria of infection were only clinical in our study as well as other studies, it may be underestimated. Although application of local antibiotics as prophylactic agents needs to be confirmed [20], we used a topical ophthalmic antibiotic as a lubricant and prophylactic agent. This may explain the lower rate of infection compared to other mentioned studies.

We had five cases (1.3%) of redundant mucosa in Plastibell group that may be due to the inappropriately sized bell. The choice of a correctly sized bell is important. If the bell is too small, it causes compression of the glands and edema, thus leading to micturition difficulty. If the bell is too large, proximal dislocation or distal dislocations can occur [9].

Although the PD group had a diverse type of complications, bleeding was the only complication of CDS group and it occurred in 1.95% of subjects.

As reported in other studies [8], an obvious advantage of using the Plastibell was the short surgery time. Average procedure duration with the PD group was 3.4 minutes, compared with 9.2 minutes with the sleeve resection.

There are a few limitations for this study. First, a variety of surgical methods are available for neonatal circumcision, and this study assessed only two of them. In addition, the higher complications with the PD compared to CDS should not be externalized to other nonsurgical approaches (i.e., Gomco clamp). This study was not a complete randomized trial. The cosmetic appearance and also the parent satisfaction were not prospectively assessed. We are planning to follow up with subjects in terms of possible long-term complications, similar to other studies [1, 12, 21].

## 5. CONCLUSION

Based on results of this study, the overall complication rate of CDS is less than that of the Plastibell method. The bell separation time directly correlates with the age and weight of infants. We suggest the Plastibell method for neonates and low-weight infants with thin prepuce, and the CDS for other infants.

## Figures and Tables

**Table 1:** Demographic characteristics of subjects.

	Conventional dissection	Plastibell Method
	surgery (*n* = 205)	(*n* = 381)
Age (m)	4.4 ± 3.2	3.1 ± 2.8
Weight (kg)	6.6 ± 2.3	5.6 ± 1.9
Upper percentile 10	2.9%	2.6%
Lower percentile 10	4.9%	5.8%

**Table 2:** Complications of circumcision by conventional dissection surgery and Plastibell methods based on the weight percentile of subjects (chi-square analysis).

Complications	Conventional dissection surgery (*n* = 205)							Plastibell method (*n* = 381)							*P*-value (surgery versus Plastibell)
	Upper 10%		Normal		Lower 10%		*P*-value*	Upper 10%		Normal		Lower 10%		*P*-value*	.051
	weight		weight		weight			weight		weight		weight			
	(*n* = 6)		(*n* = 189)		(*n* = 10)			(*n* = 10)		(*n* = 349)		(*n* = 22)			
	N	%	N	%	N	%		N	%	N	%	N	%	
Infection	0	0	0	0	0	0	.162	0	0	4	1.05	0	0	.935	
Bleeding	0	0	3	1.46	1	0.49		0	0	5	1.31	0	0		
Hematoma	0	0	0	0	0	0		0	0	1	0.26	0	0		
Excess mucosa	0	0	0	0	0	0		0	0	4	1.05	1	0.26		
Disposition	0	0	0	0	0	0		0	0	2	0.53	0	0		
Delayed falling	0	0	0	0	0	0		1	0.26	8	2.10	1	0.26		
No complication	6	2.93	186	90.73	9	4.39		9	2.36	325	85.3	20	5.25		

*P-value of complications in each group based on their weight percentile.
